# Electrophysiological Signatures of Intrinsic Functional Connectivity Related to rTMS Treatment for Mal de Debarquement Syndrome

**DOI:** 10.1007/s10548-018-0671-6

**Published:** 2018-08-11

**Authors:** Yoon-Hee Cha, Guofa Shou, Diamond Gleghorn, Benjamin C. Doudican, Han Yuan, Lei Ding

**Affiliations:** 1 Laureate Institute for Brain Research, 6655 South Yale Avenue, Tulsa, OK 74136 USA; 2 University of Tulsa, Tulsa, OK USA; 3 Stephenson School of Biomedical Engineering, University of Oklahoma, Norman, OK USA; 4 Institute for Biomedical Engineering, Science and Technology, University of Oklahoma, Norman, OK USA

**Keywords:** rTMS, Mal de debarquement syndrome, Intrinsic functional connectivity, EEG, Inter-independent component phase coherence

## Abstract

To determine intrinsic functional connectivity (IFC) related to symptom changes induced by rTMS in mal de debarquement syndrome (MdDS), a motion perceptual disorder induced by entrainment to oscillating motion. Twenty right-handed women (mean age: 52.9 ± 12.6 years; mean duration illness: 35.2 ± 24.2 months) with MdDS received five sessions of rTMS (1 Hz right DLPFC, 10 Hz left DLPFC) over consecutive days. High-density (128-channel) resting-state EEG were recorded prior to and following treatment sessions and analyzed using a group-level independent component (IC) analysis. IFC between 19 ICs was quantified by inter-IC phase coherence (ICPC) in six frequency bands (delta, theta, low alpha, high alpha, beta, gamma). Correlational analyses between IFCs and symptoms were performed. Symptom improvement after rTMS was significantly correlated with (1) an increase in low alpha band (8–10 Hz) IFC but a decrease of IFC in all other bands, and (2) high baseline IFC in the high alpha (11–13 Hz) and beta bands (14–30 Hz). Most treatment related IFC changes occurred between frontal and parietal regions with a linear association between the degree of symptom improvement and the number of coherent IFC changes. Frequency band and region specific IFC changes correlate with and can predict symptom changes induced by rTMS over DLPFC in MdDS. MdDS symptom response correlates with high baseline IFC in most frequency bands. Treatment induced increase in long-range low alpha IFC and decreases in IFC in other bands as well as the proportion of coherent IFC changes correlate with symptom reduction.

## Introduction

Mal de debarquement Syndrome (MdDS) is a motion-triggered disorder of persistent oscillating vertigo that occurs after prolonged exposure to passive motion (Bisdorff et al. 2009; Brown and Baloh 1987). As opposed to motion sickness that occurs during the motion exposure, MdDS occurs after the motion stimulus has ended (Golding 2016). It represents the consequence of the human brain’s entrainment to external oscillating motion such as during sea travel, the most common trigger (Cha 2009; Hain et al. 1999). The hallmark feature of MdDS includes a perception of rhythmic self-motion described as rocking, bobbing, or swaying that is only relieved with re-exposure to passive motion (Hain and Cherchi 2016; Hain et al. 1999; Cha et al. 2008; Cha 2009). While brief episodes of post-motion exposure dizziness and vertigo lasting less than two days are common, persistent episodes of MdDS lasting for months or years can occur in an important minority of individuals (Cha 2012; Hain et al. 1999; Cha et al. 2008; Stoffregen et al. 2013; Gordon et al. 2000; Nachum et al. 2004).

Sensory entrainment to motion that does not revert back to the native state has neuroimaging signatures (Ding et al. 2014; Yuan et al. 2017; Cha et al. 2012). Functional neuroimaging of the MdDS brain state has revealed increased metabolism in the left entorhinal cortex and amygdala, two brain areas that exhibit entrainment to periodic electrical stimulation (Cha et al. 2012; Egorov et al. 2002, 2006; Fransén et al. 2006). Enhanced functional connectivity between the entorhinal cortex and posterior parietal and occipital areas that serve visual, vestibular, and somatosensory integration functions has been noted in MdDS (Cha et al. 2012).

Repetitive transcranial magnetic stimulation (rTMS) over the dorsolateral prefrontal cortex (DLPFC) is able to acutely reduce the perception of oscillating self-motion in MdDS (Cha et al. 2013). Connectivity between the entorhinal cortex and the posterior default mode network decreases with symptom improvement after rTMS over DLPFC (Ding et al. 2014; Yuan et al. 2017; Cha et al. 2012). Laterality plays a role in the optimal treatment paradigm in MdDS with right-handed subjects responding better to left DLPFC 10 Hz stimulation and left-handed subjects responding better to right DLPFC 10 Hz stimulation. Additionally, right 1 Hz stimulation in right-handed subjects also confers benefits (Cha et al. 2013). These findings are consistent with rTMS studies over the DLPFC in depression in which both high frequency (10 Hz) over the left DLPFC and low frequency (1 Hz) over the right DLPFC confer anti-depressant effects (Liu et al. 2017; Donse et al. 2018). The parallel is relevant because depression scores are higher in MdDS than in healthy controls and there may be synergistic benefit to both motion perception and mood obtained by targeting a shared circuitry (O’Reardon et al. 2007; Liao et al. 2011; Arroll et al. 2014).

Resting-state intrinsic functional connectivity (IFC) is a term used to denote baseline connectivity that is not stimulus driven (Van Dijk et al. 2009). It may be quantified as inter-independent component phase coherence (ICPC) on high-density EEG, which has previously been shown to correlate with symptom status in MdDS after bilateral DLPFC stimulation (Ding et al. 2014). Posterior predominant ICPC reductions occur in individuals with MdDS who are successfully treated with rTMS over the DLPFC (Shou et al. 2014; Ding et al. 2014). A model for MdDS has been proposed in which limbic oscillators that have widespread functional connectivity are tuned by the periodic stimulation of passive motion exposure that, in the presence of a relative reduction of prefrontal control over these limbic areas, causes a maladaptive physiological state to become persistent (Dickson et al. 2000; Cha 2015). Notably, MdDS may recur spontaneously after remission, indicating a training effect or memory of a maladaptive state in some cases. In the right physiological background, this might occur spontaneously without a motion trigger (Cha et al. 2008, 2018; Cha and Cui 2013).

MdDS represents a natural model of sensory entrainment to environmental motion that may provide new insights into the human brain’s strategy for encoding periodic motion. We present IFC changes in a larger group of MdDS patients with a more thorough analysis method than our prior studies using the same rTMS paradigm (Ding et al. 2014; Shou et al. 2014). We have additionally determined baseline IFC characteristics that correlate with symptom improvement. We also present novel findings regarding the characterization of the IFCs as predictors of treatment effects, as well as the IFCs that simultaneously act as predictors and indicators of treatment effects.

## Materials and Methods

### Informed Consent

Study procedures were completed according to Declaration of Helsinki guidelines and approved by Western IRB (http://www.wirb.com). Participants provided written informed consent and were recruited under ClinicalTrials.gov study NCT02470377. Details of the long-term treatment effects have been previously reported (Cha et al. 2016). This study used rTMS in an off-label manner (not used for the currently FDA approved indications for depression or peripheral nerve stimulation).

### Inclusion and Exclusion Criteria

Inclusion criteria included: (1) chronic perception of rocking vertigo that started within two days after disembarking from sea, air, or land based travel; (2) symptoms lasting at least 6 months; (3) no other cause for symptoms after evaluation by a neurologist or otolaryngologist with appropriate testing for peripheral inner ear or other central nervous system cause for symptoms. Exclusion criteria included: (1) an unstable medical or psychiatric condition, including a history of bipolar disorder or psychosis; (2) pregnant or planning to become pregnant during the study; (3) contraindications to receiving rTMS or MRI, including medications known to reduce seizure threshold; (4) an unclear history of the onset of symptoms; (5) an inability to complete all study related testing.

### MRI Imaging

All participants underwent structural brain MRI imaging for use in neuronavigation during the rTMS sessions. A T1-weighted magnetization-prepared rapid gradient-echo (MPRAGE) sequence with SENSE using the following parameters was acquired: FOV = 240 mm, axial slices per slab = 190, slice thickness = 0.9 mm, image matrix = 256 × 256, TR/TE = 5/2.012 ms, acceleration factor R = 2, flip angle = 8°, inversion time TI = 725 ms, sampling band-width = 31.2 kHz.

### rTMS Procedures

rTMS was performed with the Magventure MagPro X100 stimulator with a cooled figure-of-eight coil in biphasic mode with the handle back at a 45-degree angle relative to the mid-sagittal plane. The Localite TMS Navigator (Localite GmBH, Germany) frameless stereotaxy system was used for neuronavigation to identify the center of the DLPFC as the anterior portion of the middle of the middle frontal gyrus. Motor thresholds (MT) were determined each day with independent measurements made for both the right and left M1 hand areas before each of five treatment sessions that were performed on consecutive days at the same time each day. MTs were defined as the percent intensity of the stimulator output that generated a 50 µV motor evoked potential in the contralateral *abductor pollicis brevis* muscle in 50% of trials. rTMS sessions consisted of 1 Hz right DLPFC stimulation at 110% of MT for 1200 pulses followed by 10 Hz left DLPFC stimulation at 110% MT for 2000 pulses. The 10 Hz protocol was administered as trains of 40 pulses over 4 s (10 Hz) followed by 26 s of rest.

### Symptom Measures

The participants rated their symptom status on a visual analogue scale (VAS) of 0–100 in which 0 represented complete absence of rocking vertigo and 100 represented symptoms so severe that they could not remain upright. The change of the VAS score from the first (Day 1) to the last (Day 5) day was calculated. Participants whose symptoms decreased by 10-points or more were considered to be “positive” responders (improved); those whose scores increased by 10 points or more were considered “negative” responders (worsened); those with scores in-between were considered to be “neutral.” The minimum criterion of 10 points was chosen because it best correlated with the participants’ own perceptions of significant symptom change.

### EEG Recording and Preprocessing

Two sessions of 5-min resting state EEG data acquisition were performed before the first rTMS session (Day 1) and about 3 h after the last rTMS session (Day 5) using a 128-channel EEG cap (Brain Products GmbH, Munich, Germany). The post rTMS EEG data were acquired with a delay because of the obligatory time spent to prepare the cap on the subject with electrode paste. The post-treatment EEG data thus represented more sustained neuromodulatory effects than if the recordings were made immediately following the last treatment. The online reference channel was located at the FCz position and the ground electrode at the AFz position. Impedance of all electrodes was maintained below 10 KΩ with a sampling frequency of 1000 Hz, an analog filter from 0.016 to 250 Hz, and a resolution of 0.1 µV. Participants sat in a chair with their eyes closed in a quiet darkened room during the recording.

The preprocessing steps for each participant were fully detailed in Ding et al. 2014. To briefly review, data were sequentially processed as follows: (1) notch filtered at 60 Hz, (2) band-pass filtered at 0.5–100 Hz, (3) noisy channels removed, (4) segmented into one second epochs without overlap from continuous recordings, (5) noisy segments removed, (6) decomposed remaining data into 64 ICs via independent component analysis (ICA) implemented in EEGLAB (Delorme and Makeig 2004), (7) noisy ICs removed using the *FASTER* toolbox and visual inspection (Nolan et al. 2010). An average of 284 epochs were left after preprocessing for each recording session, which were further down-sampled to 250 Hz and re-referenced to a common average.

### Detection of IFCs at the Component Domain

IFCs were measured at the component domain rather than the channel domain in order to reduce volume conduction effects on channel signals. First, a group-level ICA using a complex ICA model with a real-value mixing matrix was performed on the preprocessed EEG data that were concatenated across all sessions (Ding et al. 2014; Bingham and Hyvärinen 2000; Shou et al. 2012). The input data, complex values corresponding to 2–50 Hz with a resolution of 1 Hz, was obtained by short-time Fourier transform. Second, brain activity related components were selected from all obtained components based on their spatio-spectral patterns for subsequent analysis (Ding et al. 2014). Third, IFC between different components, quantified as ICPC (Delorme and Makeig 2004), which is also known as phase locking value (Lachaux et al. 1999), was measured at six different frequency bands, i.e., delta (2–3 Hz), theta (4–7 Hz), low alpha (8–10 Hz), high alpha (11–13 Hz), beta (14–30 Hz), and gamma (31–50 Hz). The motivations for using ICPC were (1) phase coherence takes nonlinearity into account (Van Diessen et al. 2015), and (2) the calculation of phase coherence between independent components rather than channels has been shown to largely reduce the volume conduction effect in the estimation of functional connectivity (Shou and Ding 2015).

### Identification of Treatment-Related IFC.

To identify rTMS treatment-related IFCs, four different analyses were sequentially performed on ICPC values between different components.

1. Pre- and post-rTMS ICPC values were separately examined via a one-sample *t* test to determine whether they were significantly different from zero (null hypothesis). The significance level was set to *p* < 0.05 with Bonferroni correction [significance level divided by the number of IC pairs based on 19 selected ICs (i.e., 19*(19 − 1)/2) * the number of frequency bands (i.e., 6)].
2. Cross-subject Pearson correlation analysis was performed on the pre- to post-rTMS (i.e., post-rTMS minus pre-rTMS) ICPC differences and VAS scores to identify symptom change-related IFCs. An IFC was of statistical significance only when the *p* value of the correlation analysis was < 0.05 and the ICPC value was significantly different from zero in either the pre- or post-rTMS session, as detected in the first analysis.
3. An independent cross-subject Pearson correlation analysis was performed between pre-rTMS (i.e. baseline) ICPC values and VAS score changes to identify IFCs whose baseline values correlated with treatment response. Here, an IFC was considered statistically significant only when the *p* value of the correlation analysis was < 0.05 and the ICPC value was significantly different from zero in the pre-rTMS session detected in the first analysis.
4. Based on the overlapping IFCs that showed consistently significant correlations in both the prior correlation analyses, we calculated the proportion of IFCs that exhibited the same direction (coherency) of changes induced by rTMS as indicated in the prior correlation analyses for each participant. A third cross-subject correlation analysis was then conducted between this proportion and the VAS score changes. The goal of this analysis was to examine the treatment-related IFC changes beyond individual IFCs in order to determine whether there was a general pattern of IFC changes that correlated with treatment response.

In steps 2 and 3, we did not perform a correction for multiple comparisons in terms of the number of IC pairs and frequency bands, but only implemented a constraint that the ICPC values were different from zero in either pre- or post-rTMS session.

## Results

### Demographic Information

**Fig. 1:**
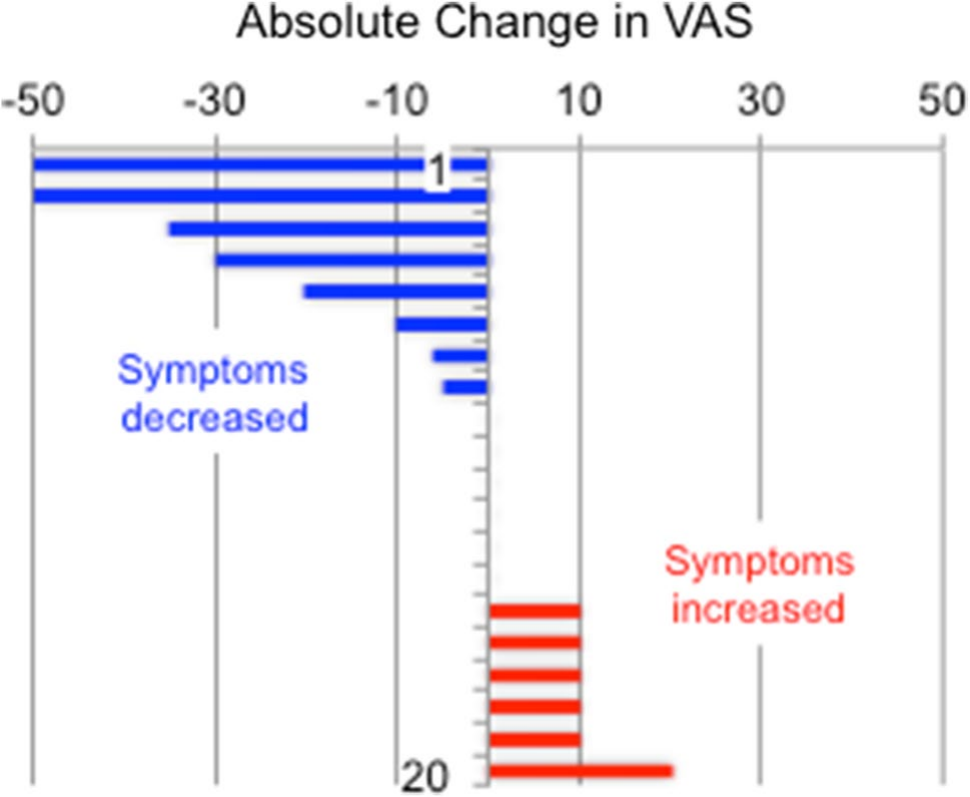
Change in visual analogue scores (VAS) following rTMS

Twenty right-handed individuals with MdDS completed all 5 days of the protocol. Despite being open to either sex, only female participants volunteered for the study, reflecting the significantly higher proportion of women to men in this disorder (Cha et al. 2018; Mucci et al. 2018). Mean age at the time of the study was 52.9 ± 12.6 years, median of 56.5 years, and a range of 28–68 years. The duration of illness had a mean of 35.2 ± 24.2 months, median 30 months, and a range of 8–96 months. Inciting triggers included water-based travel in 11, air travel in four, and land-based (car, train) travel in five. Changes of VAS scores from Day 1 to Day 5 are presented in Fig. 1.

### Components of Interest

**Fig. 2:**
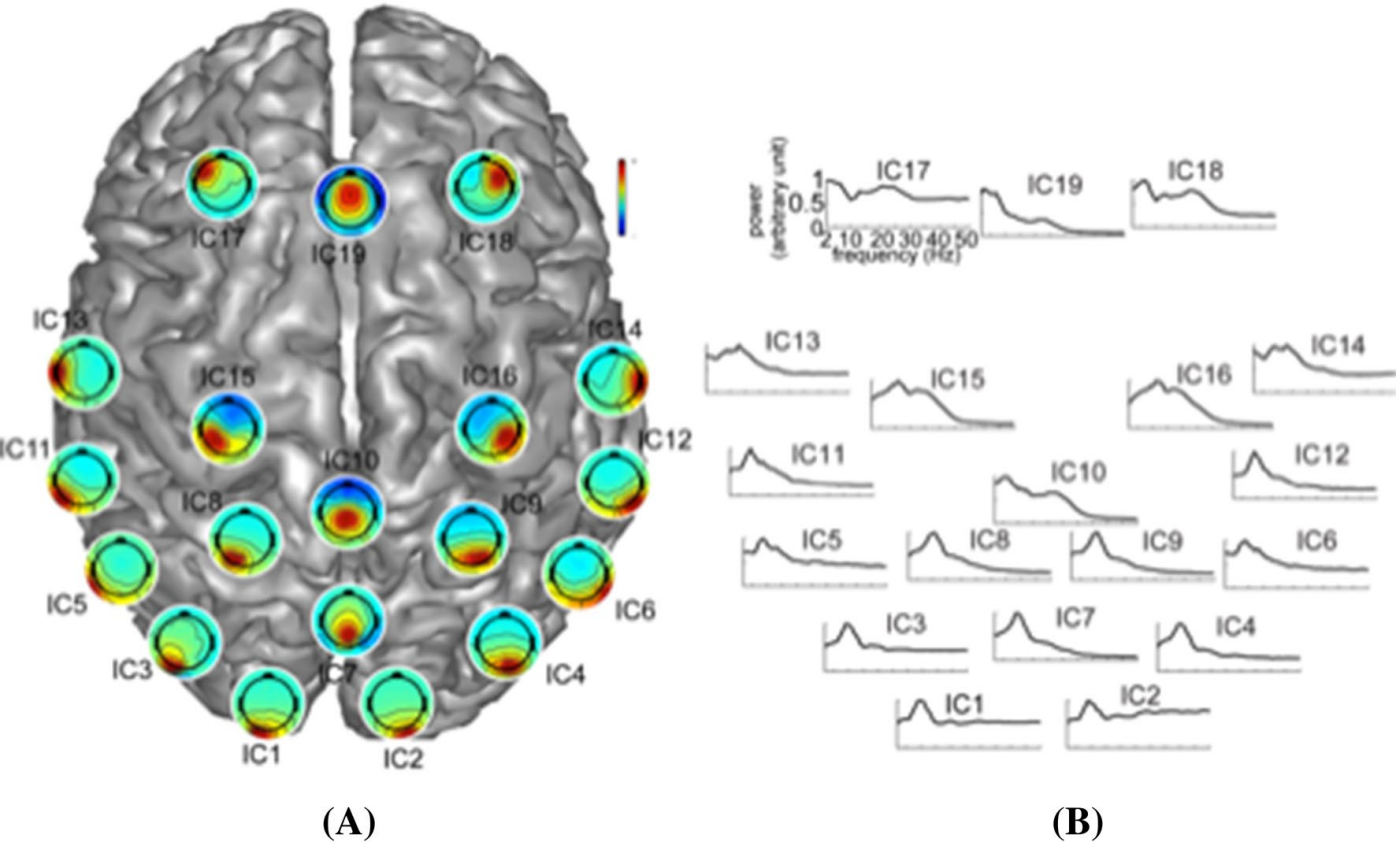
Spatial and spectral patterns of 19 selected independent components arranged over the cortex according to the approximate locations of their neural substrates: **A** spatial patterns, **B** normalized grand averaged power spectra from 2 to 50 Hz

Nineteen ICs were selected based on their spatial and spectral characteristics (labeled as IC*k, k* = 1…19, Fig. 2). These components generally replicated the IC patterns of many other studies as well as our previous studies using resting-state EEG signals (Ding et al. 2014; Yuan et al. 2012; Chen et al. 2013). These ICs are displayed on a cortex model based on the dominant weights of their spatial patterns in Fig. 2A. Most of the ICs are located symmetrically on both hemispheres or along the midline of the cortex well-representing most brain regions, i.e., ICs 1–4 for occipital, ICs 7–10 for parietal, ICs 5–6, 11–14 for tempo-occipital/temporal, ICs 15–16 for motor, and ICs 17–19 for frontal regions. IC spectral patterns (Fig. 2B) followed the 1/f distribution in their power spectra with peaks at corresponding frequency bands, i.e., peaks at alpha band for most ICs, and additional peaks in theta and beta bands for the ICs over frontal and parietal regions. Such phenomena were consistent with the regional specificity of the dominant frequency band power over different brain regions (Shou et al. 2012; Klimesch 1999).

### IFC as an Indicator of Treatment Effect

**Fig. 3:**
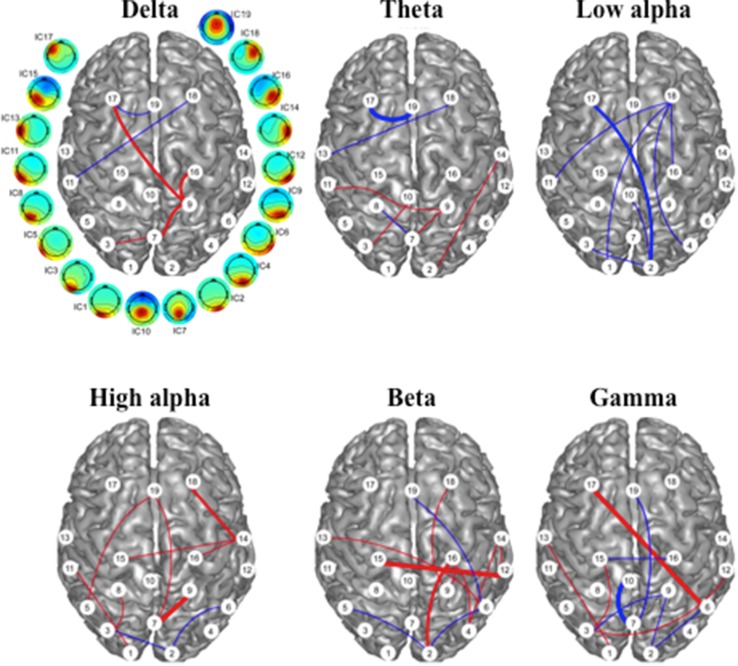
Intrinsic functional connectivity changes correlated to VAS score change following rTMS. Red and blue lines indicate positive and negative correlations, respectively. Red: connectivity decreases with symptom improvement; blue: connectivity increases with symptom improvement. The weight of the lines represents levels of significance: heavy: *p* < 0.005, medium: *p* < 0.01, light: *p* < 0.05. White circles with numbering indicate the ICs noted in Fig. 2

Intrinsic functional connectivity changes that significantly correlated with VAS score changes are illustrated in Fig. 3. In the low frequencies (i.e., delta, theta, and low alpha), there were 6, 7, and 7 IFCs identified, respectively. In the high frequencies (i.e. high alpha, beta, and gamma), there were 11, 11, and 10 identified. The IFC changes in the low alpha band were all *negatively* correlated (positive vs. negative = 0 vs. 7), indicating that rTMS-induced IFC enhancement in this band was correlated with a reduction in symptoms. Conversely, the IFCs in the high alpha and beta bands were predominantly positive (positive vs. negative = 9 vs. 2 for high alpha, and 8 vs. 3 for beta), indicating a general rTMS-induced IFC reduction in the high alpha and beta bands with improvement in symptoms. The IFCs in other frequency bands showed no predominant directional correlation with symptom change. When counting the ICs that were identified in these IFCs across all frequency bands, six ICs were present at least once in five frequency bands, i.e., IC2, IC3, IC9, IC16, IC18 and IC19, and five ICs were present in at least eight IFCs across all frequency bands, i.e., IC2(11), IC3(8), IC7(8), IC9(10), and IC18(8). It was notable that most of the ICs and IFCs that correlated with symptom changes were long-range and inter-hemispheric, especially related to frontal and parietal regions.

### IFC as a Predictor of Treatment Effect

**Fig. 4:**
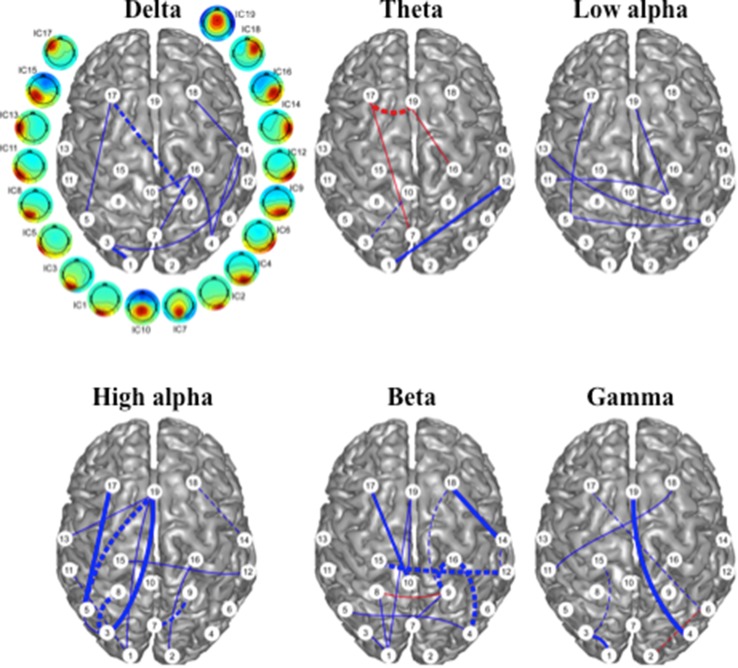
Intrinsic functional connectivity (IFC) with baseline values significantly correlated with VAS score changes following rTMS. Red: low baseline connectivity correlated with symptom improvement; blue: high baseline connectivity correlated with symptom improvement. Dashed lines indicate IFCs that were identified in both the symptom correlation analysis shown in Fig. 3 and in the baseline correlation analysis. The weight of the lines represents levels of significance: heavy: *p* < 0.005, medium: *p* < 0.01, light: *p* < 0.05. White circles with numbering indicate the ICs noted in Fig. 2

Baseline IFC measurements that were significantly correlated to VAS score changes and may be predictive of treatment response are illustrated in Fig. 4. Among the six frequency bands, 9, 13, and 13 IFCs were identified in the delta, high alpha, and beta bands, respectively. There were five IFCs identified in each of the theta, low alpha, and gamma bands. Most of the detected IFCs had negative correlations, indicating that, in general, high pre-rTMS functional connectivity was correlated with treatment response to rTMS. Regarding the involved ICs, four ICs were present at least once in five or more frequency bands, i.e., IC1, IC3 and IC19 in five bands, and IC17 in six bands. Five ICs were present in 8 or more IFC measurements in all six-frequency bands, i.e., IC1, IC9, IC17 in 8 IFCs, IC3 in 9 IFCs, and IC19 in 10 IFCs. Similarly, these ICs were mainly in the frontal and posterior (parietal and occipital) regions, and most of the identified measures of IFC were involved in long-range and inter-hemispheric connections.

### IFC with Both Predictive and Dynamic Changes Related to Treatment Effect

A group of IFCs were identified with significant correlations in the preceding two types of correlation analyses. Among them, 16 IFCs were simultaneously identified with consistent correlation patterns (Fig. 4), i.e., they both changed with treatment response and were also predictive of treatment response. Among different frequency bands, most were detected in the high alpha and beta bands (i.e., 5), while no common IFCs were detected in the low alpha band. It is also notable that most of these common IFCs (14 out of 16) had high pre-rTMS values and negative changes (i.e. connectivity went down) corresponding to lowering VAS (symptom improvement).

**Fig. 5:**
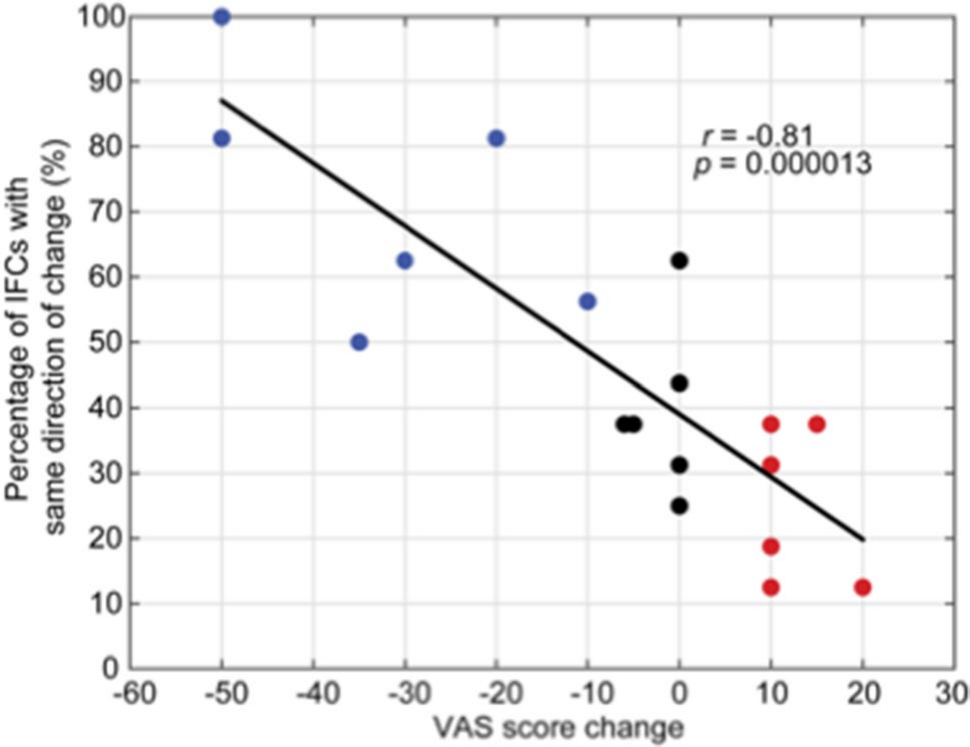
Relationship between all simultaneously detected IFCs across all frequency bands (dashed lines in Fig. 4) and treatment effects for all subjects: blue, black and red dots represent positive, neutral and negative responders, respectively

The correlation analysis between the coherency of IFCs changes (exhibiting the same direction of change) and VAS score changes revealed a significantly negative relationship (*r* = − 0.81, *p* = 0.000013, Fig. 5). This indicated that individuals with more IFCs exhibiting the same direction of change induced by rTMS (i.e., usually a positive correlation signifying a reduction in IFC with a reduction in symptoms) were more likely to be positive responders. Specifically, positive responders had at least 50% of common IFCs across all frequency bands exhibiting the same direction of change, whereas neutral/negative responders all had less than 50% of common IFCs exhibiting the same direction of change. This indicated that synchronization is a relevant factor in the pathogenesis of MdDS and specifically that de-synchronization of IFCs correlate with symptom improvement.

## Discussion

The present study investigated electrophysiological signatures of rTMS treatment-related IFCs in individuals with MdDS by examining pre- and post-rTMS resting-state EEG signals and their correlation to symptom change. These findings provide a detailed evaluation of IFC changes induced by rTMS beyond those revealed in a pilot study (Ding et al. 2014). Frequency- and region-specific IFCs were found to be modulated after rTMS with changes significantly correlated to symptom change, especially in the low/high alpha and beta bands in the frontal and posterior regions. In addition, IFCs that predicted an individual’s response to rTMS were identified, with high baseline values predictive of a positive treatment effect. More importantly, some IFCs that correlated with symptom change also overlapped with those predictive of symptom change.

The correlation analysis between IFC change and symptom change revealed a frequency band-dependent pattern (Fig. 3). Among six frequency bands, positive and negative correlations were dominant in the high alpha/beta bands, and low alpha, respectively. The different patterns for low and high alpha bands might be related to different neural mechanisms in these alpha frequency sub-bands (Klimesch 1999; Klimesch et al. 1993).

Alpha oscillation is traditionally referred to as an “idling rhythm,” and its fluctuation indicates either disengagement (high power) or engagement (low power) of specific neurocognitive processes (Klimesch et al. 2007; Klimesch 1999). Lower alpha band synchronizations and de-synchronizations are widespread and reflect general demands on attention (Klimesch et al. 1998; Neuper and Pfurtscheller 2001). The negative correlation of the low alpha band to symptom change (i.e. connectivity goes up when symptoms go down) may represent the unloading of the attentional demand created by the motion perception following rTMS. In contrast, upper alpha band power has been reported to decrease over the motor cortex during physical movement, visual cortex during visual stimulation, and temporal cortex during auditory stimulation (Chatrian et al. 1959; Berger 1929; Niedermeyer 1990). It has been proposed that upper alpha amplitude is associated with the inhibition of non-essential cognitive processing, allowing greater efficiency of top-down control over the task at hand (Klimesch et al. 2007; Cooper et al. 2003; Bazanova and Vernon 2014). Therefore, the reduced connectivity of high alpha band following rTMS could be correlated with less rhythmic idling and better engagement of both cognitive and external sensory processing.

Compared to other frequency bands, the functional role of beta oscillations remains controversial (Engel and Fries 2010). The identified pattern of dominant positive correlation in the beta band might be interpreted by the proposed “status quo” hypothesis (Engel and Fries 2010), which suggests that beta activity is associated with the maintenance of the present sensorimotor or cognitive state with its reduction indicating a change of the status quo. Alternatively, a second recently proposed theory for the role of beta-synchronization suggests that it may mediate the transition from latent to active cortical representations, which may be content specific (Spitzer and Haegens 2017). According to this theory, the beta band may be a “transitional” rhythm that bridges the gap between alpha frequency, which is associated with top-down inhibition (Klimesch et al. 2007) and gamma frequency (30–100 Hz), which is associated with local activity (Whittingstall and Logothetis 2009). Changes in connectivity in the beta band, therefore, may reflect an alteration in the ability to reactivate latent endogenous activity.

The correlation analysis in this study on pre-TMS values revealed a pattern of mostly negative correlations among all six bands (Fig. 4), indicating that individuals with higher baseline values achieved better treatment outcomes. More importantly, some IFCs were detected in both correlation analyses indicating that they had both predictive and dynamic features (Fig. 4), i.e., treatment responders were individuals with high baseline IFC values that reduced following rTMS. These data were consistent with high baseline resting state functional connectivity between DLPFC and entorhinal cortex correlating with treatment response to rTMS in MdDS (Yuan et al. 2017). The DLPFC is also the main target for rTMS treatment of medically refractory depression, with a resultant large body of literature on the neuroimaging correlates of DLPFC stimulation for depression (Perera et al. 2016; Fischer et al. 2016). In particular, the therapeutic effect by targeted stimulation at DLPFC has been linked to a modulatory effect in the circuitry of the default mode network (Liston et al. 2014). These hubs include the frontal and posterior regions observed in the current study. Furthermore, Drysdale et al. (2017) reported that intrinsic network connectivity is predictive of TMS treatment response for depression with key regions involved in predicting responders including the DLPFC and posterior parietal cortex. This is again consistent with our observations, despite being based on different neuroimaging modalities. In contrast, anti-correlation between DLPFC and subgenual anterior cingulate cortex has been shown to predict treatment response to rTMS for depression, indicating that specific networks may exhibit oppositely directed dynamic changes related to symptom response (Fox et al. 2012).

To further examine the correlation between IFCs and treatment effect, we performed a third correlation analysis (Fig. 5). The results indicated that not only individual IFCs but also the *proportion* of dynamic IFC changes across all frequency bands is a marker of treatment response. Specifically, responders to rTMS exhibited a greater proportion of IFC de-synchronizations, regardless of frequency. Therefore, these common IFCs as well as the number of IFCs could be biomarkers used to both predict and monitor treatment effects. Thus, a goal of treatment for MdDS might be to induce a greater proportion of IFC de-synchronizations.

Past studies have shown that functional connectivity to latent drivers of neural oscillators such as the entorhinal cortex is altered between individuals with MdDS and healthy controls, which may explain the widespread IFC changes observed in this study (Cha et al. 2012; Yuan et al. 2017). The components involved in the detected IFCs were mainly in the frontal and posterior regions. Though we targeted the DLPFC in this study, we have shown that induced IFC changes were indeed widespread, and were directional for those in whom a beneficial clinical effect was induced. IC2, IC3, and IC7 in the occipital and posterior parietal regions and IC17 and IC18 in the prefrontal regions were the most commonly observed locations that exhibited symptom related connectivity changes. This is especially relevant because the target of the treatment was the prefrontal cortex in the region of ICs 17 and 18, whereas symptom relevant visual and motion processing areas are in the regions of ICs 2, 3, and 7. This raises the possibility that some posterior nodes could serve as potential stimulation targets for future studies.

The current results of IFC based on EEG data are related to previous investigations in resting-state functional connectivity using fMRI, particularly with regard to the default mode network changes (Yuan et al. 2016; Raichle et al. 2001; Buckner et al. 2008). EEG can reveal frequency-specific connectivity changes with a stronger relationship between certain frequency bands and symptom changes than others. A decrease in resting state fMRI connectivity in the posterior default mode network that correlated with symptom improvement after DLPFC stimulation in individuals with MdDS (Yuan et al. 2017) was consistent with the decrease of EEG connectivity in the delta, high-alpha, beta band and gamma bands observed in this study, indicating a positive coupling relationship between the fMRI data and resting-state EEG. Notably, the positive coupling in the beta and gamma frequency bands has been observed from simultaneous EEG and fMRI resting-state data as well as local field potential and fMRI data in other human studies (Mukamel et al. 2005; Mantini et al. 2007; Laufs et al. 2003).

When examining regions where EEG connectivity is associated with positive coupling with fMRI data, our results show near-range connections in the parietal and occipital regions as well as long-range connections between the parietal/temporal cortices and the medial frontal regions, hub regions of the default mode network. Indeed, consistent spatial patterns between EEG and fMRI resting-state networks have been confirmed in recent studies that report a high concordance between the spatial and temporal dynamics of the default mode network (Yuan et al. 2012, 2013, 2016). Our current study reports an opposite trend in the low alpha frequency band as well as theta band in the frontal regions (Fig. 3), indicating that symptom reductions are associated with EEG connectivity increases in these lower frequency bands. Compared with fMRI findings, the increase of EEG connectivity in the low-alpha frequency band coincides with a decrease of fMRI connectivity, suggesting a negative coupling relationship. Our observation of the negative coupling in the low-alpha frequency band with resting state fMRI is consistent with other human brain studies (Mukamel et al. 2005; Laufs et al. 2003; Goldman et al. 2002; de Munck et al. 2007).

### Limitations

Several study limitations merit comment. For the purposes of our analyses, though an even distribution of treatment responses created an input variable on which we could determine symptom related IFC changes, the low number of individuals in each category could have lead to inappropriate weighting of small amounts of data. Mitigating this somewhat is that participants with positive responses (improvement) showed IFC changes discrete from those who either did not change or who worsened, creating basically two functional groups—responders and non-responders. Supporting this pattern is that these IFC results were consistent with resting-state functional connectivity changes measured with BOLD, which also showed that those who improve with rTMS exhibit connectivity patterns different from those who either show no change or who worsen (Yuan et al. 2017).

Second, we did not use an active control for rTMS. This could have been helpful in determining random differences in IFC due to the passage of time. However, as we learned from a prior analysis, IFC measurements made pre- and post-rTMS without respect to symptom changes show no consistent patterns; they appeared to be random (Ding et al. 2014). Only IFC changes binned according to treatment response groups show any coherent relationship, as in our current analyses. In both these EEG and prior fMRI analyses, we required that IFC changes be directionally related to symptom changes, indicating that the study was internally controlled (if connectivity went up for one direction of symptom change, it was required to go down for the opposite symptom change). For ethical reasons, we could not give repetitive sessions of rTMS to healthy control participants in order to determine intrinsic long-term rTMS effects.

Third, no explicit correction of multiple comparisons in terms of the number of IC pairs and frequency bands was applied in the correlation analyses in steps two and three. Each IC represents signals from strongly synchronized neural activity generated by neural substrates covering distributed brain regions (Delorme et al. 2012). Therefore, the analyses on ICs automatically involve application of a spatial cluster-based correction for multiple comparisons (Maris and Oosternveld 2007), given that EEG signals at each channel reconstructed from each IC will have the same statistics as the IC statistic. However, it does not address correction for multiple comparisons across IC pairs and frequency bands. In future studies with more subjects, multiple comparisons correction should be implemented to further verify the identified patterns.

Finally, though the use of ICA has improved spatial specificity of neural activities compared to channel signal, it does not reveal detailed information about the identity of the underlying neural substrates that generate the signal. Advances in cortical source imaging techniques may better correlate electrophysiological signatures of IFCs with cortical structures (Yuan et al. 2016; Liao et al. 2012; Zhu et al. 2014; Ding 2009). In the current analyses, we were only able to identify the general region of the affected signal. However, symptom related long-range connectivity differences were sufficient to identify frequency specific fronto-parietal and interhemispheric connectivity changes relevant to symptom status that have already been informative in subsequent neuromodulation trials (NCT02540616).

## Conclusion

Our study revealed the electrophysiological signatures of IFC underlying symptom changes induced by rTMS treatment in MdDS. Symptom improvement after rTMS was significantly correlated with IFCs that had predominantly reduced values in all frequencies with the notable exception of the low alpha band (8–10 Hz), in which IFC increased after successful treatment. A favorable response was associated with higher baseline IFC values and a greater coherence in the direction of IFC change induced by rTMS. Though this was true in all frequency bands, the strongest correlations were in the high alpha and beta bands. In addition, most of these IFCs covered frontal and parietal regions with long range and interhemispheric connectivity. Uncovering these prognostic and treatment related factors may aid in the development of more precise and effective neuromodulation therapies for motion perception disorders, including studies that aim to prime the brain by enhancing IFC and by targeting specific frequency bands for treatment.
